# Constructing Morphologically Tunable Copper Oxide-Based Nanomaterials on Cu Wire with/without the Deposition of Manganese Oxide as Bifunctional Materials for Glucose Sensing and Supercapacitors

**DOI:** 10.3390/ijms23063299

**Published:** 2022-03-18

**Authors:** Han-Wei Chang, Song-Chi Chen, Pei-Wei Chen, Feng-Jiin Liu, Yu-Chen Tsai

**Affiliations:** 1 Department of Chemical Engineering, National United University, Miaoli 360302, Taiwan; chen31423@yahoo.com.tw (S.-C.C.); vi890902@gmail.com (P.-W.C.); liu@nuu.edu.tw (F.-J.L.); 2 Pesticide Analysis Center, National United University, Miaoli 360302, Taiwan; 3 Department of Chemical Engineering, National Chung Hsing University, Taichung 402202, Taiwan

**Keywords:** morphologically tunable copper oxide-based nanomaterials, non-enzymatic glucose sensing, supercapacitor, heterostructured Mn-Cu bimetallic oxide architectures

## Abstract

Morphologically tunable copper oxide-based nanomaterials on Cu wire have been synthesized through a one-step alkali-assisted surface oxidation process for non-enzymatic glucose sensing. Subsequently, copper oxide-based nanomaterials on Cu wire as a supporting matrix to deposit manganese oxide for the construction of heterostructured Mn-Cu bimetallic oxide architectures through spontaneous redox reaction in the KMnO_4_ solution for supercapacitors. Field emission scanning electron microscopy (FESEM), field emission transmission electron microscopy (FETEM), X-ray diffraction (XRD), and X-ray photoelectron spectroscopy (XPS) confirmed that morphological and phase transformation from Cu(OH)_2_ to CuO occurred in copper oxide-based nanomaterials on Cu wire with different degrees of growth reaction. In non-enzymatic glucose sensing, morphologically tunable copper oxide-based nanomaterials owned the high tunability of electrocatalytically active sites and intrinsic catalytic activity to meet efficient glucose electrooxidation for obtaining promoted non-enzymatic glucose sensing performances (sensitivity of 2331 μA mM^−1^ cm^−2^ and the limit of detection of 0.02 mM). In the supercapacitor, heterostructured Mn–Cu bimetallic oxide-based nanomaterials delivered abundant redox-active sites and continuous conductive network to optimize the synergistic effect of Mn and Cu redox species for boosting the pseudo-capacitance performance (areal capacitance value of 79.4 mF cm^−2^ at 0.2 mA cm^−2^ current density and capacitance retention of 74.9% after 1000 cycles). It concluded that morphologically tunable copper oxide-based nanomaterials on Cu wire with/without deposition of manganese oxide could be good candidates for the future design of synergistic multifunctional materials in electrochemical techniques.

## 1. Introduction

Most global burdens (substantial human diseases and global warming) are linked to public health and environmental problems that may negatively impact human health and economic development. Thus, effective creative strategies could open up new opportunities to ensure health promotion/disease prevention and environmental impact reductions. Noncommunicable diseases (principally cardiovascular diseases, chronic respiratory diseases, cancers, and diabetes) are major global public health problems, which lead to a large global public health burden [1]. In 2016, the World Health Organization (WHO) reported that noncommunicable diseases were the leading cause of all death worldwide at 71.0%, and a high proportion of deaths for 26.3% occurred at a premature age (aged 30–70 years) [2]. In order to improve the health and wellbeing of the world, Sustainable Development Goal (SDG) target 3.4 aims to achieve the goal of a one-third reduction of premature mortality from noncommunicable diseases (aged 30–70 years) by 2030. Present studies also reported that people’s unhealthy eating behavior and lifestyle increased the potential influence of metabolic abnormalities, and predicted an increasing risk of noncommunicable diseases, including obesity, hyperglycemia, dyslipidemia, and high blood pressure, which consequently led to glucose metabolic diseases (type 2 diabetes mellitus, non-alcoholic fatty liver, atherosclerosis, and encephalopathy) [3,4]. In fact, many mechanisms of physiological/pathophysiological functions are tightly linked to glucose metabolism. Thus, the ability of glucose monitoring has a positive impact on the control and prevention of noncommunicable diseases related to glucose metabolism.

On the other hand, environmental awareness and natural resources exhaustion force to look towards renewable energy resources for clean and sustainable energy development, and mitigate environmental problems and natural resources consumption. Supercapacitors, as an important component of sustainable energy conversion and storage devices with properties that are intermediate between traditional capacitors and batteries, exhibit excellent performance at simple construction, short charging time, safety, high power density, and excellent lifetime, which are believed to be useful for reducing the fossil fuel consumption and further relaxing environmental concerns [5,6].

It has been demonstrated that electrochemical techniques with moderate cost, instrumental simplicity, high portability, and suitable performance show its fascinating application in solar cells, batteries, sensors, and supercapacitors, and offer an excellent solution to address public health, environmental and sustainable energy requirements. The rational design of electrode materials and the construction of novel electrode structures have been regarded as prospective strategies to build the modeling and optimization of electrochemical devices in future electrochemical applications.

Electrode materials made with three dimensional (3D) interconnected conductive network architectures exhibit a large electric contact surface of the active materials available by the electrolyte, having the ability to change the electrochemical properties of the electrode materials, and thus effectively accelerate electron/ion delivery through multiple interconnected conductive network architectures, which lead to enhance the kinetic behavior at the electrochemical interface impacted by electrochemical reactions. The presented 3D interconnected conductive network strategies in metal-based materials would offer the effectiveness of this approach to the combination of the structural interconnectivity and the outstanding properties, and thus make them a promising candidate to prepare conductive substrate as high performance electrode material in the future synthesis for the application in electrochemical techniques [7,8]. The metal-based materials (Cu, Ni, Fe, Co, Mn, etc.) with a well controllable procedure and synthesis through in situ nucleation and growth processes can be used as an ideal platform for the growth of transition metal oxides/hydroxides including CuO, Cu(OH)_2_, Co_3_O_4_, Co(OH)_2_, NiO, Ni(OH)_2_, MnO_x_, FeO_x_, etc. [9,10,11,12]. It could not only develop porous structural interconnectivity supply additional accessible space for minimizing the ionic and electronic transportation distances but also improve bonding strength between metal network nanostructure and select electrode materials to prevent delamination during processing. It is further expected to offer much improved electrochemical performances of the electrodes. Among metal-based materials, Cu oxide-based materials possess attractive properties, such as rich abundance, inexpensiveness, low resistance, electrocatalytic activity, environmental compatibility, and controlled morphology, which are further expected to offer much improved electrochemical performances [13,14]. The morphology controlled Cu oxide-based materials (nanoflowers, nanorods, nanosheets, nanowires, etc.) could be applied in the modification of electrode materials and have great influences on the electrochemical performance [15,16], which could provide a novel electrochemical platform for the construction of non-enzymatic glucose sensing in practical applications.

In addition to being used as an electrode material for non-enzymatic glucose sensing, Cu oxide-based materials also stimulate more research enthusiasm for a supercapacitor, which can be properly combined with other mono transition metal oxides to form binary transition-metal oxides with multidimensional hierarchical structure. This has the potential to achieve the goal of obtaining a higher performance of electrode materials for supercapacitors. To the best of our knowledge, manganese oxide has great advantages in terms of excellent capacity, low cost, natural abundance, and multiple crystallographic phases to be one of the most promising candidates in supercapacitors [17,18]. Therefore, the obtained optimum balance between the excellent capacity of manganese oxide and good electrical conductivity of Cu oxide-based materials is expected to exhibit improved electrochemical performance when being used in supercapacitors due to the synergistic effect.

In this work, we report a method for the controlled synthesis of morphologically tunable copper oxide-based nanomaterials on Cu wire that have the potential to modulate the electrocatalytic activity toward glucose oxidation reaction in alkaline solution for non-enzymatic glucose sensing. Subsequently, we directly construct copper oxide-based nanomaterials on Cu wire as a supporting matrix to deposit manganese oxide for building supercapacitors. Constructing morphologically tunable copper oxide-based nanomaterials on Cu wire with/without deposition of manganese oxide as bifunctional materials display a significant improvement of electrochemical performance to meet the ever-increasing requirements in glucose sensing and supercapacitors.

## 2. Materials and Methods

### 2.1. Reagents

Potassium permanganate (KMnO_4_), sodium hydroxide (NaOH), ammonium persulfate ((NH_4_)_2_S_2_O_8_), glucose, and potassium hydroxide (KOH) were purchased from Sigma-Aldrich (St. Louis, MO, USA). All water used was deionized water (DI water) through Milli-Q water purification system (Millipore, MA, USA). All chemicals were used without further purification.

### 2.2. Synthesis of Morphologically Tunable Copper Oxide and Heterostructured Mn-Cu Bimetallic Oxide-Based Nanomaterials on Cu Wire

A series time-dependent morphological evolution of copper oxide-based nanomaterials on Cu wire were synthesized through one-step alkali-assisted surface oxidation process. First, the Cu wires were ultrasonically cleaned 3 times in ethanol and DI water to remove the surface oxidation layer. After that, cleaned Cu wires were immersed into a mixture of aqueous solution (14 mL) of 5 M NaOH and 0.25 M (NH_4_)_2_S_2_O_8_ at room temperature by controlling the reaction time of 10, 20, and 40 min (low, medium, and high level reactivity), subsequently, washed 3 times with DI water and dried at room temperature in air. With the low, medium, and high level of reactivity in alkaline solution, the rod-like, mixture of rod-like/sheet-like, and sheet-like of morphologically tunable copper oxide-based nanomaterials could be formed on Cu wire by regulating reaction time. The obtained morphologically tunable copper oxide-based nanomaterials with rod-like, mixture of rod-like/sheet-like, and sheet-like morphologies on Cu wire were denoted as Cu-R, Cu-M, and Cu-S, respectively. Then, the obtained Cu-R, Cu-M, and Cu-S were washed with deionized water to remove the remaining reagents and collected for subsequent manganese oxide synthesis. Manganese oxide was spontaneously deposited on the Cu-R, Cu-M, and Cu-S by spontaneous redox reaction [18]. The synthesis procedure were as follows: the obtained Cu-R, Cu-M, and Cu-S were immersed in 120 mL of aqueous solutions of 0.005 M KMnO_4_ with stirring of the solution at 85 °C for 1 h. Subsequently, these obtained products (heterostructured Mn–Cu bimetallic oxide-based nanomaterials) were rinsed 3 times by DI water and dried at 60 °C for 3 h (denoted as named MnCu-R, MnCu-M, and MnCu-S, respectively). And then the resulted MnCu-R, MnCu-M, and MnCu-S were collected for the following characterization.

### 2.3. Apparatus

The morphology was characterized by using field emission scanning electron microscopy (FESEM, JSM-7410F, JEOL, Tokyo, Japan) and field emission transmission electron microscopy (FETEM, JEM-2100F, JEOL, Tokyo, Japan). The chemical structure and composition was determined by X-ray photoelectron spectroscopy (XPS, PHI-5000 Versaprobe, ULVAC-PHI, Chigasaki, Japan). X-ray diffraction (XRD) pattern was recorded using a D8 Discover (Bruker) X-ray diffractometer with Cu Kα radiation. Electrochemical measurements were performed using a three-electrode system by an electrochemical analyzer (Autolab, model PGSTAT30, Eco Chemie, Utrecht, The Netherlands). For three electrode system, the assembly approach of electrodes were direct growth of morphologically tunable copper oxide and heterostructured Mn–Cu bimetallic oxide-based nanomaterials on Cu wire and were directly used as the working electrode without any polymer binder or conductive additives, platinum wire was used as the counter electrode, and Ag/AgCl (3 M KCl) was used as the reference electrode. The electrochemical testing were performed in the two different aqueous electrolyte solutions as supporting electrolyte (using 0.1 M NaOH for electrochemical glucose sensing and 1 M KOH for supercapacitor).

## 3. Results

**Figure 1:**
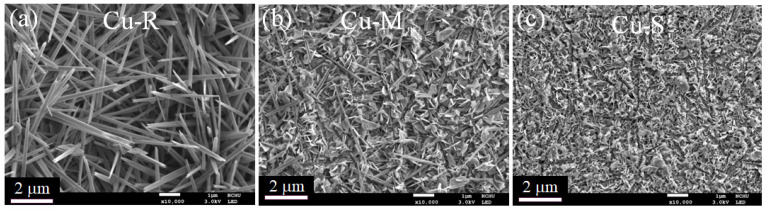
FESEM images of (**a**) Cu-R, (**b**) Cu-M, and (**c**) Cu-S.

**Figure 2:**
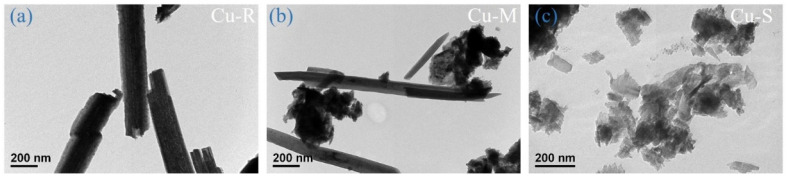
FETEM images of (**a**) Cu-R, (**b**) Cu-M, and (**c**) Cu-S.

The morphologies of Cu-R, Cu-M, and Cu-S are characterized by FESEM and FETEM. FESEM images in Figure 1 display the time-dependent morphological transformation of Cu-based materials. The higher-magnification FESEM images of these samples are shown in Figure 1a–c. A dramatically morphological variation from rod-like to sheet-like shape of copper oxide-based nanomaterials occurs while controlling the reaction time (low, medium, and high level reactivity), which exhibits a significant time-dependent morphological evolution. The morphological evolution of Cu-based materials is further characterized in detail using FETEM. FETEM images (Figure 2) clearly show the changes in the morphological evolution of Cu-based materials when controlling the reaction time. Figure 2a reveals that the TEM image of low level reactivity in Cu-based material has rod-like morphology with an average diameter of about 200 nm and an average length of about 2 μm. By increasing the reaction time from low to medium level reactivity, it seems that the morphological transition from one-dimensional rod-like to two-dimensional sheet-like morphologies is usually performed in a mixture of rod-like/sheet-like morphologies, and these two-dimensional sheets are covered by the clusters with a mean lateral length of several tens of nanometers, which are consistent with the FESEM images in the Figure 1. As the reactions continue to achieve high level reactivity, Cu-based materials confirm the complete morphological transition of sheet-like morphologies, which fairly densely and uniformly grow on the entire surface of Cu wire (Figure 1). The observed mean lateral length of sheets in FETEM reaches several tens of nanometers (see Figure 2c). It can expect that the morphological tunability of the structure in Cu-based materials can be considered the practical use for many future electrochemical applications.

**Figure 3:**
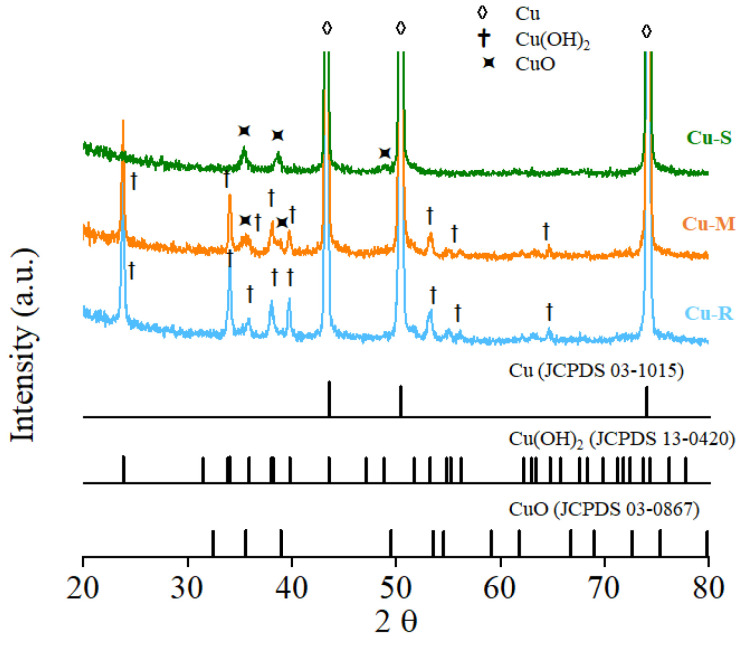
XRD patterns of Cu-R, Cu-M, and Cu-S.

The structure and phase composition of Cu-R, Cu-M, and Cu-S are characterized by XRD (Figure 3). A clear crystalline phase transformation with the time-dependent morphological transformation could be revealed from the comparison of the XRD patterns. The identification of the crystalline phases are carried out by standard Joint Committee on Powder Diffraction Standards (JCPDS) cards available in the system software for the presence of Cu (JCPDS Data Card No. 03-1015), Cu(OH)_2_ (JCPDS Data Card No 13-0420), and CuO (JCPDS Data Card No 03-0867) as major components (marked with ◊, †, and 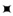 signs). Obviously, three strong diffraction peaks at 2θ of 43.3 (111), 50.4 (200), and 74.3 (220) in the three Cu-based samples belong to the metallic Cu, which are attributed to the Cu wire substrate [19]. The results show that the XRD pattern of Cu-R obtains peaks at 2θ of 23.9, 34.0, 35.9, 38.1, 40.0, and 53.3 corresponding to (021), (002), (111), (022), (130), and (150) planes, matching the JCPDS file for Cu(OH)_2_ [20]. With the increase of reaction time, the crystalline phase transformation of Cu(OH)_2_ to CuO is found to be at the XRD pattern of Cu-M. The additional diffraction peaks are observed at 2θ of 35.7, 39.0, and 49.5 corresponding to (−111), (111), and (−202) planes [21], which are ascribed to the formation of CuO. Notice that the Cu(OH)_2_ phase is mostly transformed into CuO phase, as indicated in the XRD patterns of Cu-S, meaning an almost complete phase transformation from Cu(OH)_2_ to CuO via the time-dependent driving force of the phase transformation under alkaline condition, which occurs mainly by a continuous reconstructive transformation from Cu(OH)_2_ nanorod to crystalline CuO nanosheet [22]. The XRD patterns clearly provide conclusive evidence that the crystalline phase transformation of Cu(OH)_2_ to CuO can be controlled to depend on time for alkali-assisted surface oxidation proceeding.

**Figure 4:**
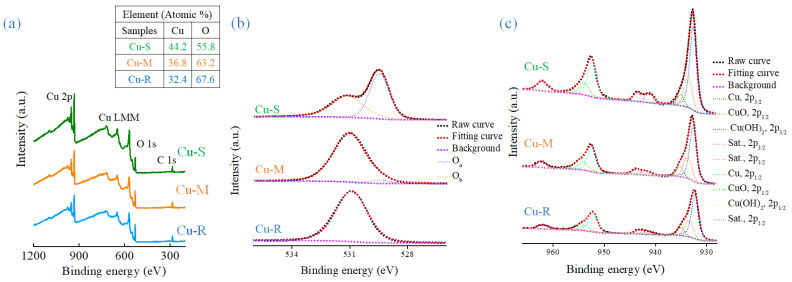
XPS spectra of Cu-R, Cu-M, and Cu-S (**a**) full survey, (**b**) O 1s, and (**c**) Cu 2p.

The surface chemical and electronic states of Cu-R, Cu-M, and Cu-S are characterized by XPS (Figure 4). Figure 4a presents the XPS full survey spectra of Cu-R, Cu-M, and Cu-S (the atomic percentages are included in the inset of Figure 4a). The results indicate the coexistence of O and Cu elements in the morphologically tunable copper oxide-based nanomaterials at different degrees of growth reaction. The high-resolution O 1s XPS spectra of Cu-R, Cu-M, and Cu-S are shown in Figure 4b. The O 1s XPS spectra can be divided into two main peaks (O_a_ and O_b_) located at about 529.5 and 531.0, which correspond to metal-oxide (CuO) and metal hydroxide (Cu(OH)_2_), respectively [23]. The O 1s XPS quantitative analysis results are summarized in Table 1 and it confirms Cu(OH)_2_ to CuO phase transformation in time-dependent morphological transformation of Cu-based materials. The high-resolution Cu 2p XPS spectra of Cu-R, Cu-M, and Cu-S are shown in Figure 4c. The spectra show spin-orbit doublets of Cu 2p_3/2_ and Cu 2p_1/2_ located at about 934.2 and 954.4 eV with spin–orbit energy separation of about 20 eV, concluding that the Cu^2+^ chemical state is presented as Cu(OH)_2_ and/or CuO in the oxidized form [24]. Furthermore, the overlapped Cu 2p_3/2_ (2p_1/2_) spectra can be fitted into three peaks at 932.3 (952.2), 933.5 (953.8), and 935.0 (955.0) eV, which are the characteristics of Cu, CuO, and Cu(OH)_2_. Additionally, the appearance of shake-up satellite peaks (denoted as Sat.) with the binding energy of 941.6, 943.0, and 962.0 eV at the higher binding energies of the main peaks indicate the presence of an unfilled Cu 3d^9^ shell, proving again the existence of Cu^2+^ chemical state in the samples [25]. From a deeper insight into the Cu 2p XPS quantitative analysis (Table 2), the ratio of CuO to Cu(OH)_2_ in the Cu-R, Cu-M, and Cu-S increases with the level of reactivity of the chemical transformations in alkaline solution. Based on the above analysis, it suggests that higher content of CuO is more pronounced for the sample with high-level reactivity (Cu-S), further declaring Cu(OH)_2_ and CuO phase transformation in the morphologically tunable copper oxide-based nanomaterials at different degrees of growth reaction.

**Figure 5:**
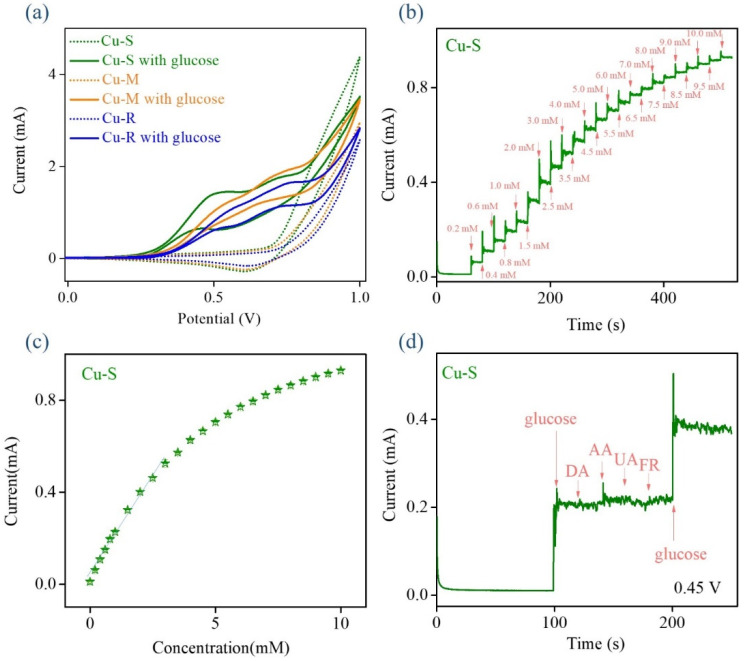
(**a**) CV curves of Cu-R, Cu-M, and Cu-S in 0.1 M NaOH in the absence (dashed lines) and presence (solid lines) of 10 mM glucose at a scan rate of 50 mV s^−1^. (**b**) Amperometry of Cu-S in 0.1 M NaOH with successive addition of various glucose concentrations at applying voltage +0.45 V. (**c**) Corresponding calibration plot of the response current versus glucose concentration. (**d**) Interference test of Cu-S in 0.1 M NaOH at applying voltage +0.45 V.

To evaluate the potential use of morphologically tunable copper oxide-based nanomaterials for electrochemical non-enzymatic glucose sensing, Figure 5 presents the electrochemical characterization results of Cu-R, Cu-M, and Cu-S thorough cyclic voltammetry (CV) and amperometry. As the results show in Figure 5a, the CV curves of Cu-R, Cu-M, and Cu-S are performed under the scan rate of 50 mV s^−1^ in 0.1 M NaOH absence and presence 10 mM glucose. In comparison with CV results, these morphologically tunable copper oxide-based nanomaterials (Cu-R, Cu-M, and Cu-S) make a difference in the electrochemical sensing properties for non-enzymatic glucose detection. The successive addition of glucose results in an increase of the anodic (oxidation) current. These results clearly suggest that Cu-S shows the highest anodic (oxidation) peak current of glucose under a relatively low-onset potential (0.45 V), revealing that Cu-S owns the strongest electro-catalytic performance towards the oxidation of glucose when compared to Cu-R and Cu-M [26]. According to the obtained results, it can be concluded that this direct-growth approach of Cu-S on Cu wire conductive substrate promotes interfacial electrons transfer and presents easily high conductivity between the electrolyte/electrode. Further, perfect crystalline phase and morphological properties of Cu-S also provides a large quantity of electrocatalytically active sites and intrinsic catalytic activity that greatly facilitates the transformation of copper redox couple during the oxidation of glucose in alkaline solution to confer them great possibilities in more efficient glucose electrooxidation for obtaining promoted glucose sensing performances [27,28]. Figure 5b shows the amperometry of Cu-S towards glucose electrooxidation in 0.1 M NaOH at 0.45 V with the gradual additions of glucose concentrations up to 10 mM. The corresponding calibration plot of the response current (I) versus glucose concentration (Conc._glu_) (Error bars represent the standard deviations for the measurements repeated three-times) is shown in Figure 5c. The response current of Cu-S increases linearly with glucose concentration up to about 3 mM. The corresponding calibration equation is presented as I (mA) = 0.18 Conc._glu_ (mM) + 0.016, and the resulting correlation coefficient value (R^2^) of 0.994, sensitivity of 2331 μA mM^−1^ cm^−2^, and the limit of detection of 0.02 mM based on a signal-to-noise ratio of 3 (S/N = 3), suggesting the improved electrocatalytic activity of Cu-S toward glucose electrooxidation in alkaline media. The relative performance (sensitivity) of Cu-S is comparable with values obtained in previous investigations of copper based materials for electrochemical non-enzymatic glucose sensing, including CuO-chitosan nanocomposite (503 μA mM cm^−2^) [29], CuO nanorod (1523.5 μA mM cm^−2^) [30], nanolayers of carbon protected copper oxide (272.6 μA mM cm^−2^) [31], CuO thin films (1311.8 μA mM cm^−2^) [32], and CuO nanosheets (520.0 μA mM cm^−2^) [33].

It is a well-known fact that there are potential interfering species commonly encountered in glucose monitoring, hence, it was needed to evaluate their effect in the presence of potential interfering species with the glucose monitoring. Naturally, the blood glucose concentration in an apparently healthy human is about 30-times higher than that of other interfering species. Therefore, in interference test, the concentration of the interfering species at a level 10-times lower than that of glucose are used in subsequent interference tests [34]. The interference test in the presence of potential interfering species is conducted by amperometry in 0.1 M NaOH at 0.45 V with the gradual additions of 1.0 mM glucose, 0.1 mM dopamine (DA), 0.1 mM ascorbic acid (AA), 0.1 mM uric acid (UA), 0.1 mM fructose (FR), and 1.0 mM glucose. It is found that there is no observable interference to the response to glucose in the presence of four potential interfering species (Figure 5d). It means that the proposed Cu-S possesses the excellent selectivity (or anti-interference effect). To further demonstrate the potential of the proposed Cu-S in practical applications, the recovery tests of glucose is carried out by adding known concentration to samples (human serum and green tea), which are then evaluated by analyzing the amperometric response to glucose by measurements repeated three-times. The results of the recovery test obtained by the proposed electrochemical method are found to be in the range of 92~108% and the obtained relative standard derivations (RSD) are no larger than 3.6%. The comparison results are given in Table 3. The whole results further prove the feasibility of the use of morphologically tunable copper oxide-based nanomaterials (particularly significant in the case of the Cu-S with high ratio of CuO to Cu(OH)_2_) to be an efficient electrode materials in potential practical use for glucose detection. By characterizing the morphologically tunable observed on the copper oxide-based nanomaterials (Cu-R, Cu-M, and Cu-S), it can be expected that unique copper oxide-based nanomaterials with tunable morphology and crystalline phase could provide a new set of growth platform to form a novel heterostructured Mn–Cu bimetallic oxide architectures, which could open another opportunity to explore its possible applications in supercapacitor.

**Figure 6:**
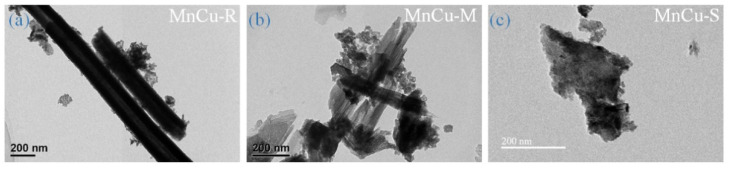
FETEM images of (**a**) MnCu-R, (**b**) MnCu-M, and (**c**) MnCu-S.

Figure 6 displays the EFTEM images of heterostructured Mn–Cu bimetallic oxide-based nanomaterials (MnCu-R, MnCu-M, and MnCu-S). Decorating manganese oxide on morphologically tunable copper oxide-based nanomaterials, it can be clearly seen in Figure 6a–c that the surface morphologies of heterostructured Mn–Cu bimetallic oxide-based nanomaterials have obvious differences when compared to copper oxide-based nanomaterials (see Figure 1). Nanosheet-shaped manganese oxide with an average size of few nanometers is decorated over copper oxide-based nanomaterials. The heterostructured Mn–Cu bimetallic oxide-based nanomaterials on Cu wire are distinctly interconnected with each other and make up an ordered heterostructure, especially in the case of MnCu-S, ensuring good electrical contact and also offering a continuous electrons pathway to facilitate ion transport. It is thus expected that the heterostructured architectures are beneficial to promote the synergistic effect of copper oxide and manganese oxide, emerging as a promising electrode material in supercapacitors.

**Figure 7:**
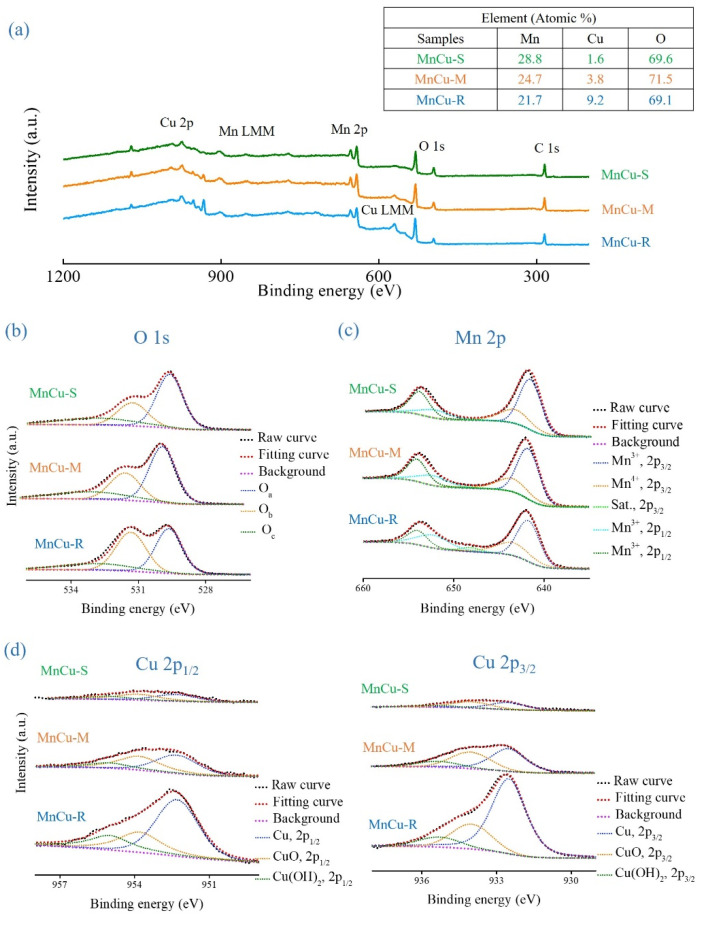
XPS spectra of MnCu-R, MnCu-M, and MnCu-S (**a**) full survey, (**b**) O 1s, (**c**) Mn 2p, and (**d**) Cu 2p_1/2_ and Cu 2p_3/2_.

In Figure 7a, the full survey XPS spectra confirm the presence of O, Cu, and Mn elements in the MnCu-R, MnCu-M, and MnCu-S (the atomic percentages are included in the inset of Figure 7a), indicating that manganese oxide is successfully synthesized on morphologically tunable copper oxide-based nanomaterials by spontaneous redox reaction. The high resolution O 1s, Cu 2p, and Mn 2p XPS spectra have been used to provide more detailed insights into the surface chemical and electronic states of MnCu-R, MnCu-M, and MnCu-S. In the high resolution O 1s XPS spectra (Figure 7b), these spectra at 529.5, 531, and 532.8 eV are deconvoluted into three peaks (O_a_, O_b_, and O_c_) corresponding to metal-oxide, metal hydroxide, and water molecule. The ratio trends in two main peaks (O_a_ and O_b_) show significant differences to that of the copper oxide-based nanomaterials (Cu-R, Cu-M, and Cu-S) (see Figure 4, Table 1 and Table 4), which could be attributed to partial overlapping with the copper and manganese valence. It is reported that O 1s XPS spectra related to the manganese oxide species mainly consist of manganese oxide (Mn–O–Mn), hydroxyl groups (Mn–O–H) in the manganese oxide [35]. The results demonstrate that the Mn valence states present in the heterostructured Mn–Cu bimetallic oxide-based nanomaterials. Notably, XPS peak O_c_ located at about 532.8 eV corresponds to the water molecule, which also confirms the presence of manganese oxide phase in the heterostructured Mn–Cu bimetallic oxide-based nanomaterials with an evidence for hydrated form [36]. In the high-resolution Mn 2p XPS spectra (Figure 7c), the Mn 2p XPS spectra of MnCu-R, MnCu-M, and MnCu-S are consisted of two doublet peaks of Mn 2p_3/2_ and 2p_1/2_ located at about 642 and 653 eV. It is found that the energy separation of the Mn 2p doublet peaks is about 11.7 eV, which can be assigned to the formation of manganese oxide in the heterostructured Mn–Cu bimetallic oxide-based nanomaterials. Furthermore, the overlapped Mn 2p_3/2_ (2p_1/2_) spectra can be fitted into three peaks at 641.9 (653.3), 643.4 (654.5), and 645.3 (657.5) eV, which are the characteristics of Mn^3+^, Mn^4+^, and shake-up satellite (denoted as Sat.), respectively, further confirming the presence of mixed-valance manganese oxide with oxidation state Mn^3+^ and Mn^4+^ [37,38]. It is concluded in agreement with the Mn 2p XPS quantitative analysis results presented in Table 5. In the high resolution Cu 2p_1/2_ and Cu 2p_3/2_ XPS spectra (Figure 7d), the overlapped Cu 2p_3/2_ (2p_1/2_) spectra can also be fitted into three peaks at 932.3 (952.2), 933.5 (953.8), and 935.0 (955.0) eV, which indicate that the copper oxide-based nanomaterials (Cu-R, Cu-M, and Cu-S) after the growth of manganese oxide retain their similar CuO/Cu(OH)_2_ ratios to that of the Cu-R, Cu-M, and Cu-S but with reduced intensity. Still, MnCu-S shows high CuO/Cu(OH)_2_ ratios. The results of Cu 2p XPS quantitative analysis are given in Table 6.

**Figure 8:**
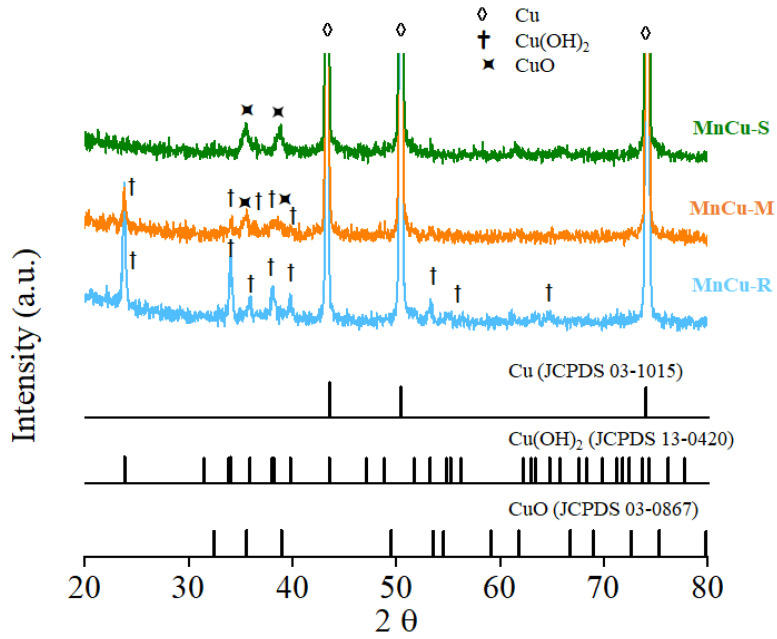
XRD patterns of MnCu-R, MnCu-M, and MnCu-S.

The structure and phase composition of MnCu-R, MnCu-M, and MnCu-S are characterized by XRD (Figure 8). The XRD analysis indicates that nanosheet-shaped manganese oxide decorating on morphologically tunable copper oxide-based nanomaterials demonstrates a similar phase transformation sequence to that of the copper oxide-based nanomaterials (Cu-R, Cu-M, and Cu-S). It is also noticeable that there is no other crystalline phase produced during manganese oxide decorating on morphologically tunable copper oxide-based nanomaterials, indicating that manganese oxide may be amorphous or has a low degree of crystallization along with crystalline copper oxide-based nanomaterials. EFTEM, XPS, and XRD clearly demonstrate that MnCu-R, MnCu-M, and MnCu-S are corresponded well to the material characteristics of copper oxide and manganese oxide, suggesting that the controlled and tunable properties of the fabrication of engineered heterostructured Mn–Cu bimetallic oxide architectures are beneficial to obtain excellent electrochemical performance characteristics in supercapacitors.

**Figure 9:**
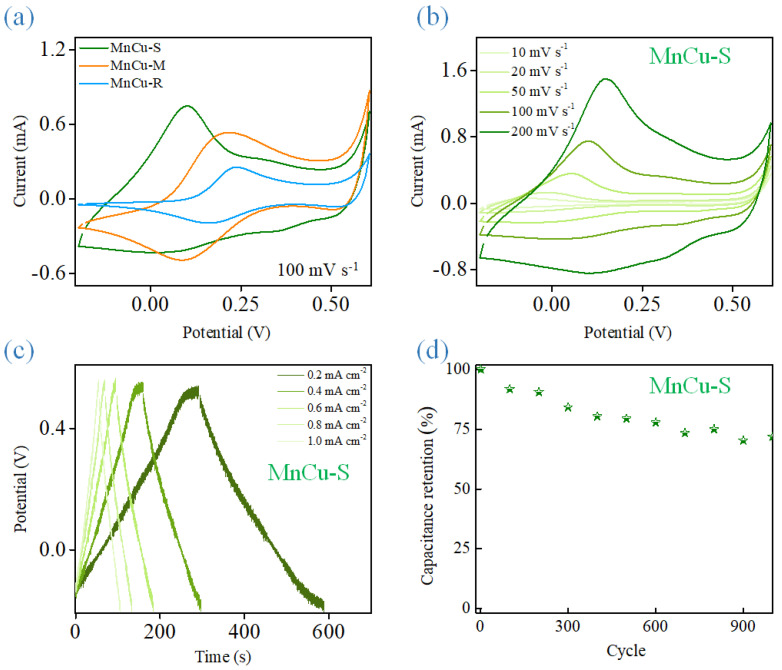
(**a**) CV curves of MnCu-R, MnCu-M, and MnCu-S (**b**) CV curves, and (**c**) galvanostatic charge/discharge curves of MnCu-S in 1 M KOH at different scan rates and current densities. (**d**) Cyclic stability of MnCu-S in 1 M KOH.

Electrochemical properties of the heterostructured Mn–Cu bimetallic oxide-based nanomaterials as supercapacitor electrodes investigated by CV and galvanostatic charge/discharge (GCD) are shown in Figure 9. Figure 9a shows the CV curves of MnCu-R, MnCu-M, and MnCu-S in a voltage of −0.2~0.6 V at scan rates of 100 mV s^−1^ in 1 M KOH. It can be seen that the distinct and asymmetry redox peaks are observed in the CV results, suggesting the pseudo-capacitance behavior corresponding to the quasi-reversible charge storage mechanism involving transition metal redox couples in the electrode materials [39]. The CV result in Figure 9a also indicates that the capacitive performance of MnCu-S is obviously better than those of MnCu-R and MnCu-M. As the scan rate increases (Figure 9b), the CV curves for the MnCu-S reveals a very rapid current response to voltage reversal at end potential and maintains pronounced peak shapes without severe distortion, revealing the ideal capacitive behaviors and good high-rate performance of MnCu-S owing to the optimal synergistic effect of Mn and Cu redox species in heterostructured Mn–Cu bimetallic oxide-based nanomaterials [40,41]. This concluded that the controllable fabrication of MnCu-S heterostructure architecture offers abundant redox-active sites and continuous conductive network to optimize electron/ion transport kinetics for boosting the capacitive performance. The GCD measurements are used to further discuss the enhanced capacitive performance of MnCu-S (Figure 9c). The shape of MnCu-S in GCD measurements is quasi-triangular at different current densities of 0.2, 0.4, 0.6, 0.8, and 1.0 mA cm^−2^, demonstrating its pseudocapacitive performance. It is also worth noticing that the broad shoulder in the charging region from 0.0 to 0.3 V is more obvious as compared to the discharging region, further confirming a quasi-reversible charge storage mechanism, which is consistent with the CV tests. The areal capacitance values can be calculated according to the following equation. C = (I × Δt)/(ΔV × A), where C is areal capacitance (mF cm^−2^), I is the charge/discharge current (mA), Δt is the discharge time (s), ΔV is the voltage change during discharge (V), and A is the area of the electrode (cm^−2^). The areal capacitance values are in the range of 79.4~66.5 mF cm^−2^ at applied current densities range of 0.2~1.0 mA cm^−2^. One thousand consecutive galvanostatic charge–discharge cycles are carried out to elucidate the long-term cycling stability of the MnCu-S at a constant current density of 10 mA cm^−2^ (Figure 9d). After 1000 cycles, 74.9% of the initial value still can be retained, demonstrating that MnCu-S maintains a good structural stability with less structural changes during long-term cycling process. The tight contact and stable interface within MnCu-S facilitate the ion migration and charge carrier trapping effects toward heterostructured Mn–Cu bimetallic oxide-based nanomaterials and Cu wire interfaces, and thus lead to an enhanced capacitance. The engineered heterostructured Mn–Cu bimetallic oxide architectures show promising features and can prove to be one of promising candidates as electrode materials for energy storage.

## 4. Conclusions

In this study, morphologically tunable copper oxide-based nanomaterials on Cu wire with/without deposition of manganese oxide as bifunctional materials displayed high electrochemical performances in multifunctional applications (including non-enzymatic glucose sensing and supercapacitors). Thanks to the morphology-controllable synthesis and crystalline phase transformation of Cu(OH)_2_ and CuO for alkali-assisted surface oxidation proceeding, it could provide a new set of growth platform to form a novel heterostructured Mn–Cu bimetallic oxide architectures. Morphologically tunable copper oxide-based nanomaterials owned the high tunability of electrocatalytically active sites and intrinsic catalytic activity to promoted non-enzymatic glucose sensing performances. Furthermore, heterostructured Mn–Cu bimetallic oxide-based nanomaterials optimize the synergistic effect of Mn and Cu redox species for boosting the pseudo-capacitance performance. Foreseeably, morphologically tunable copper oxide-based nanomaterials on Cu wire with/without deposition manganese oxide own its electrochemical synergistic effect as promising candidates for the development of electrochemical multifunctional applications.

## Figures and Tables

**Table 1:** Fitted O 1s XPS spectra results of Cu-R, Cu-M, and Cu-S.

Sample	Fitted Results of O 1s XPS Spectra	
	O_a_ (%)	O_b_ (%)
Cu-S	54	46
Cu-M	4	96
Cu-R	0	100

**Table 2:** Fitted Cu 2p XPS spectra results of Cu-R, Cu-M, and Cu-S.

Sample	Fitted Results of Cu 2p XPS Spectra									
	Cu 2p_3/2_ (%)	CuO 2p_3/2_ (%)	Cu(OH)_2_ 2p_3/2_ (%)	Sat. 2p_3/2_ (%)	Sat. 2p_3/2_ (%)	Cu 2p_1/2_ (%)	CuO 2p_1/2_ (%)	Cu(OH)_2_ 2p_1/2_ (%)	Sat. 2p_1/2_ (%)	CuO/Cu(OH)_2_
Cu-S	38.0	12.9	4.2	4.9	4.1	18.1	7.8	3.5	6.5	2.7
Cu-M	36.6	14.1	5.7	3.5	3.6	18.2	8.0	4.9	5.4	2.1
Cu-R	38.2	13.7	6.0	3.1	3.6	18.3	6.9	4.7	5.5	1.9

**Table 3:** The results of the recovery tests and relative standard derivations obtained by the electrochemical sensing and the mass spectrometric methods.

Sample	Added (mM)	Found by Electrochemical Sensing (mM)	Recovery (%)	RSD (%)
Human serum	0.48	0.44	92	3.6
Human serum	0.24	0.26	108	3.0
Green tea	0.44	0.46	105	1.4
Green tea	0.22	0.21	95	3.1

**Table 4:** Fitted O 1s XPS spectra results of MnCu-R, MnCu-M, and MnCu-S.

Sample	Fitted Results of O 1s XPS Spectra		
	O_a_ (%)	O_b_ (%)	O_c_ (%)
MnCu-S	54	25	21
MnCu-M	52	28	20
MnCu-R	42	40	18

**Table 5:** Fitted Mn 2p XPS spectra results of MnCu-R, MnCu-M, and MnCu-S.

Sample	Fitted Results of Mn 2p XPS Spectra					
	Mn^3+^ 2p_3/2_ (%)	Mn^3+^ 2p_1/2_ (%)	Sat. 2p_3/2_ (%)	Mn^4+^ 2p_3/2_ (%)	Mn^4+^ 2p_1/2_ (%)	Mn^3+^/Mn^4+^
MnCu-S	44.5	12.3	0	21.7	21.5	1.3
MnCu-M	43.8	14.5	0.6	20.4	20.7	1.4
MnCu-R	39.3	20.9	3.5	22.0	14.3	1.7

**Table 6:** Fitted Cu 2p_1/2_ and Cu 2p_3/2_ XPS spectra results of MnCu-R, MnCu-M, and MnCu-S.

Sample	Fitted Results of Cu 2p XPS Spectra									
	Cu 2p_3/2_ (%)	CuO 2p_3/2_ (%)	Cu(OH)_2_ 2p_3/2_ (%)	Cu 2p_1/2_ (%)	CuO 2p_1/2_ (%)	Cu(OH)_2_ 2p_3/2_ (%)	Cu 2p (%)	CuO 2p (%)	Cu(OH)_2_ 2p (%)	CuO/Cu(OH)_2_
MnCu-S	48.7	39.9	11.4	43.6	47.3	9.1	44.9	41.0	15.1	2.7
MnCu-M	51.1	35.8	13.1	43.3	40.1	16.6	47.3	38.0	14.7	2.6
MnCu-R	68.7	21.9	9.4	55.9	24.8	19.3	64.3	22.9	12.8	1.8

## Data Availability

Not applicable.
